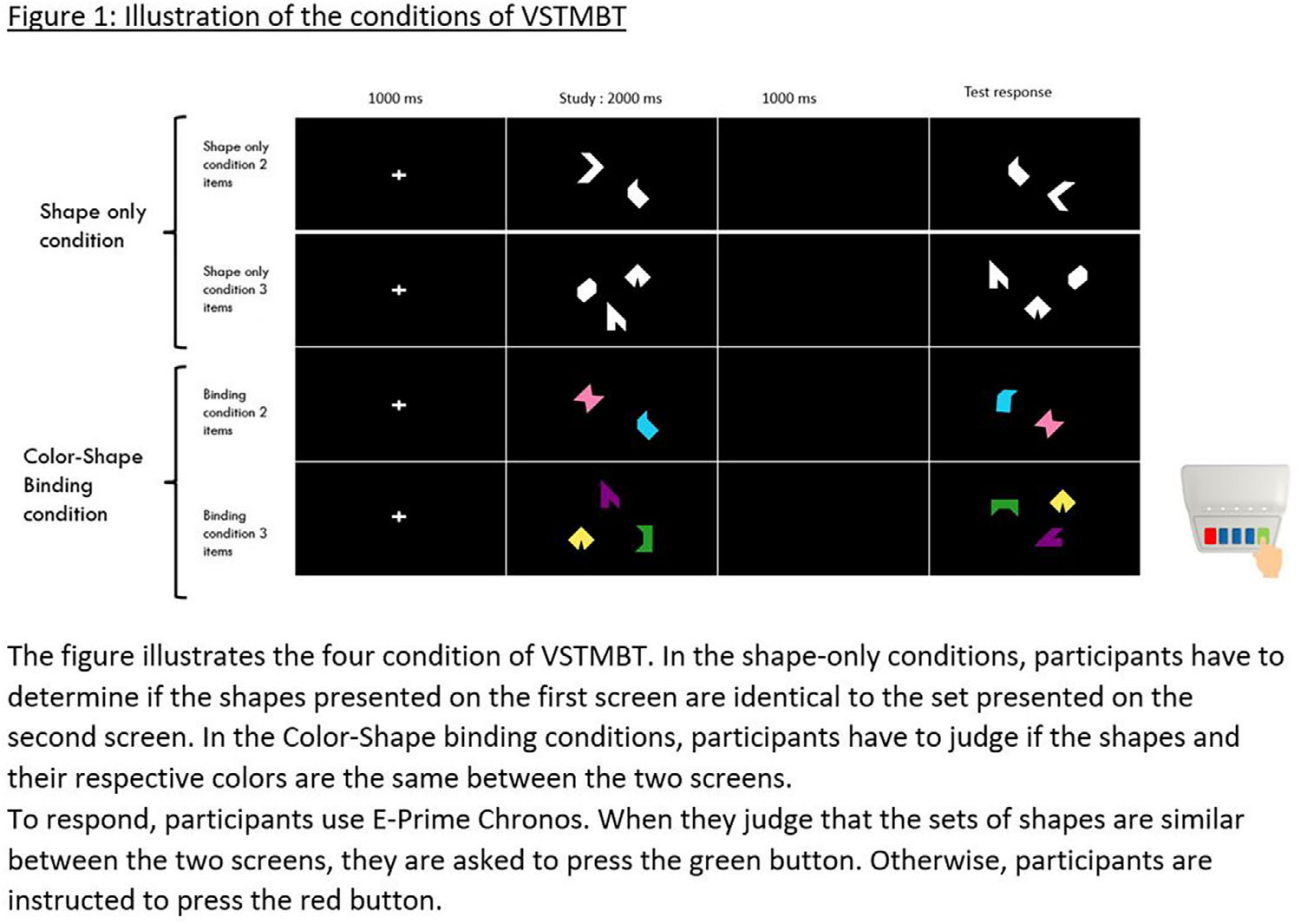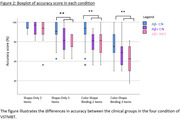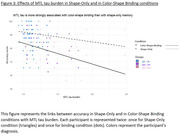# Association between short‐term memory binding performance and tau burden in the medial temporal lobe

**DOI:** 10.1002/alz.086401

**Published:** 2025-01-03

**Authors:** Lara Huyghe, Lisa Quenon, Yasmine Salman, Lise Colmant, Thomas Gérard, Vincent Malotaux, Emilien Boyer, Laurence Dricot, Renaud Lhommel, Adrian Ivanoiu, Bernard J Hanseeuw

**Affiliations:** ^1^ UCLouvain ‐ Institute of Neuroscience, Brussels Belgium; ^2^ Institute of Neuroscience, UCLouvain, Brussels Belgium; ^3^ Department of Neurology, Saint‐Luc University Hospital, Brussels Belgium; ^4^ Massachusetts General Hospital, Harvard Medical School, Boston, MA USA; ^5^ Institute of Neuroscience ‐ UCLouvain, Brussels Belgium; ^6^ Nuclear Medicine Department, Saint‐Luc University Hospital, Brussels Belgium

## Abstract

**Background:**

The ability to discriminate very similar objects by implementing the binding between their multiple features is assumed to be supported by the medial temporal lobe (MTL). MTL is the first brain region that shows abnormal tau accumulation in Alzheimer’s disease (AD). However, whether binding ability is impaired since the preclinical stage of AD and relates to MTL tau burden is not well‐established.

**Method:**

We included 35 amyloid‐negative cognitively normal individuals (Aβ‐CN), 16 amyloid‐positive CN (Aβ+CN) and 17 Aβ+ individuals with Mild Cognitive Impairment (Aβ+MCI). Aβ status was determined using lumbar punction or [^18^F]‐Flutemetamol Positron Emission Tomography (PET). Participants underwent a [^18^F]‐MK6240 tau‐PET to quantify tau burden in the MTL (entorhinal cortex, hippocampus, parahippocampus). They performed the Visual‐Short‐Term‐Memory‐Binding‐Test (VSTMBT), a test which is underpinned by the proper functioning of the MTL (Parra et al., 2010) and assess the ability to discriminate perceptually close items. In VSTMBT, participants had to determine whether a set of shapes was identical to the set presented before a 900ms blank retention interval. There were 4 conditions in which the number of shapes (2 or 3) and the color (white = ”Shape‐Only Condition” or colored) of the shapes varied. The colored conditions implied to memorize the binding between shapes and their respective color (“Binding‐Condition”)(Figure.1). We calculated the accuracy score (AS) and the mean reaction time (RT) for each condition.

**Result:**

AS was lower in both Aβ+ groups than in Aβ‐CN participants in all conditions except the Shape‐Only‐2‐items, while Aβ+CN and Aβ+MCI did not differ (Figure.2). The Aβ+CN group was slower than Aβ‐CN only in the Binding‐2‐items‐Condition.

Total AS was associated with MTL tauopathy over the entire sample (even after controlling for age and education) and when restricting to all CN participants. Finally, MTL tauopathy was more associated to AS in the Binding‐Condition than in the Shape‐Only‐Condition (Figure.3).

**Conclusion:**

Impaired visual short‐term memory performance was evidenced since the preclinical stage of AD, including in Binding‐Condition. Performance in Binding‐Condition was related to tau burden in the MTL, suggesting that impairment in visual short‐term memory binding abilities may constitute an early cognitive marker of accumulating tau pathology in the MTL.